# Uterine Fibroids and Their Association with Acute and Chronic Venous Thromboembolic Disease—An Expert Review of the Literature

**DOI:** 10.3390/jcm14124065

**Published:** 2025-06-09

**Authors:** Munaza Afaq, Brooke Alexa Zlotshewer, Estefania Oliveros, Sarah Gabrielle Bauman, Anjali Vaidya, Vladimir Lakhter, Paul Forfia, Ahmed S. Sadek, Enrique Hernandez, Riyaz Bashir

**Affiliations:** 1 Department of Cardiovascular Diseases, Temple University Hospital, Philadelphia, PA 19140, USA; mafaq@wyckoffhospital.org (M.A.); estefania.oliverossoles@tuhs.temple.edu (E.O.); anjali.vaidya@tuhs.temple.edu (A.V.); vladimir.lakhter@tuhs.temple.edu (V.L.); paul.forfia@tuhs.temple.edu (P.F.); ahmed.sadek@tuhs.temple.edu (A.S.S.); 2 Department of Medicine, Temple University Hospital, Philadelphia, PA 19140, USA; brooke.zlotshewer@temple.edu; 3 Charles E. Krausz Library, Temple University School of Podiatry Medicine—TUSPM, Philadelphia, PA 19107, USA; sarah.bauman@temple.edu; 4 Department of Obstetrics and Gynecology, Temple University Hospital, Philadelphia, PA 19140, USA; enrique.hernandez2@tuhs.temple.edu

**Keywords:** uterine fibroids, chronic thromboembolic pulmonary hypertension (CTEPH), venous thromboembolism, acute DVT, acute PE

## Abstract

Venous thromboembolism is significantly affected by hormonal and reproductive factors that pose unique challenges in women. Among various risk factors, the role of uterine fibroids, which are the most common benign tumors in women, is not well understood. The relationship between venous thromboembolism and fibroids is mainly attributed to the physical compression caused by large fibroids on pelvic veins, particularly the iliac veins, leading to venous stasis and thrombosis. This review explores the prevalence, pathogenesis, risk factors, possible racial influences, and management strategies of venous thromboembolism associated with fibroids. It highlights the need for better awareness, considering the asymptomatic nature of many fibroids and their potential to lead to serious thromboembolic complications. There is a clear need for screening methods, detailed guidelines, and treatments to prevent such complications and improve women’s health care.

## 1. Introduction

**Figure 1:**
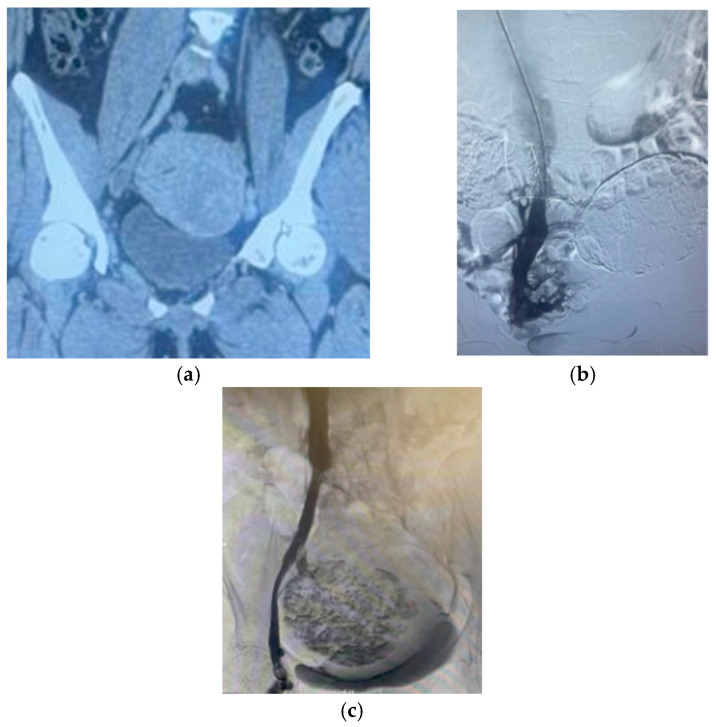
(**a**) CT Venogram showing a large uterine fibroid compressing the right external iliac vein complicated by DVT. (**b**) Patient with a large calcified uterine fibroid causing DVT and chronic occlusion of the left iliac veins. (**c**) Pelvic venogram in a patient with CTEPH showing large calcified uterine fibroid causing right external iliac compression.

Uterine fibroids are benign tumors originating from the myometrium of the uterus and are highly prevalent in women of reproductive age [1]. They are also called uterine leiomyomas or myomas. Due to their asymptomatic nature, accurately determining the prevalence of fibroids is challenging, with estimates spanning from 4.5% to 68.6% of women by age 50 [2]. The prevalence increases with age, peaking in women in their 40s. These tumors can manifest as abnormal uterine bleeding, infertility, pain, pressure symptoms, urinary symptoms, and recurrent miscarriages [3]. Additionally, uterine fibroids have been associated with venous thromboembolism (VTE), which can cause life-threatening complications [4]. Several cases of chronic thromboembolic pulmonary hypertension (CTEPH) (Figure 1c) have also been associated with fibroids, underscoring the need for increased awareness and enhanced management strategies in these patients [5].

Studies have reported varying incidences of VTE in women. Some report no significant gender-based differences in VTE, while others note a higher incidence in women, especially those under the age of 45. In these studies, the male-to-female incidence ratio ranges from 0.8 to 1.3. An increased incidence of VTE in women might be linked to pregnancy and other factors unique to their reproductive years [6]. Although fibroids are rarely reported as a direct cause, several challenges complicate the accurate assessment of their association with VTE. The asymptomatic nature of fibroids, combined with a lack of screening protocols and delayed treatment seeking, can result in missed diagnoses and insufficient monitoring. As a result, untreated fibroids may grow large enough to compress veins and increase the risk of VTE [4]. VTE in women more commonly presents as a pulmonary embolism (PE) compared to men [7], which makes this association more important to validate and confirm. Also, managing VTE in the presence of symptomatic fibroids (e.g., abnormal uterine bleeding) or during surgery for fibroids introduces unique therapeutic challenges. Since treatments for VTE can increase bleeding risks from fibroids or their surgery, it necessitates careful consideration to balance the risks and benefits of treatment options [4].

The current understanding of the association of VTE with uterine fibroids primarily stems from case reports, case series, and a limited number of observational studies. Given the paucity of comprehensive sources of data, the actual incidence of VTE in women with fibroids remains to be determined [8]. There is a lack of consensus on the screening, diagnosis, and management strategies of fibroid-associated VTE. To address this knowledge gap and improve patient care, this review aims to consolidate the existing literature and provide a narrative review of the frequency, underlying mechanisms, risk factors, and management of VTE associated with fibroids.

## 2. Review of Observational Data, Including Case Series and Case Reports

### 2.1. Material and Methods

A comprehensive literature search was conducted by a medical librarian to identify relevant studies published from database inception to September 2023. The search strategy included databases like PubMed, Embase, Web of Science, and Google Scholar. A total of 886 studies were initially identified. Studies examining an association between uterine fibroids and venous thromboembolism (VTE), including deep vein thrombosis (DVT) and/or pulmonary embolism (PE) and other kinds of venous thromboses, were included. The types of studies were case reports, case series, and observational studies (retrospective or prospective), published in English language, reporting original patient data, clinical outcomes, or mechanistic insights. Conference abstracts, editorials, letters to the editor without original data, non-peer-reviewed articles, and studies without full-text access were excluded. Case reports and case series available only as abstracts were included if they contained complete original patient data. In contrast, observational studies presented solely in abstract form were excluded due to insufficient methodological detail and incomplete data. After screening and applying inclusion criteria, the final review included three single-center and one population-based retrospective observational study. Additionally, nine case series and 57 case reports were included, encompassing a total of 100 patients. The key characteristics and findings from these studies are summarized in Table 1 and Table 2 [9,10,11,12,13,14,15,16,17,18,19,20,21,22,23,24,25,26,27,28,29,30,31,32,33,34,35,36,37,38,39].

### 2.2. Patient Demographics

The observational studies reported a mean age ranging from 45 to 64 years [40,41,42,43], while the mean age was 43.6 years in the case reports and series. One study suggested that women at younger ages may be at a higher risk of fibroid-associated VTE. It also showed a lower risk of VTE in women with fibroids aged 45 years and older (OR: 0.498, 95% CI: 0.29–0.84, *p* = 0.009) [41]. However, other studies have found no significant difference by age [43]. Approximately 25 to 40% of the patients were in the perimenopausal age group (45–55 years), suggesting a potential alteration in the VTE risk associated with fibroids following menopause [42]. Race was documented in only 35 cases, and 22 (63%) were identified as being of African descent.

### 2.3. Clinical Presentation

A study of 6095 women with uterine fibroids identified that 2% of them had VTE [40]. The most common presentation of fibroid-associated VTE in the case reports was lower extremity deep venous thrombosis (DVT) (Figure 1a), which affected the proximal venous system (popliteal, femoral, iliac veins, or inferior vena cava), accounting for 38% of the patients. This was followed by the combined presentation of DVT and PE (27.6%), with 63% of these cases presenting with proximal DVT. Six percent of the cases featured acute PE without DVT. Additional details on the other presentations are provided in Table 2. There have been some reports of ovarian vein thrombosis (OVT) [44,45,46,47]. Paradoxical emboli were reported in nine (9.2%) patients, seven of whom were found to have a patent foramen ovale (PFO). Phlegmasia cerulea dolens was reported in four (4%) patients [48,49,50,51], with two progressing to compartment syndrome [50,51]. A proportion of 63% of the DVT cases were left-sided, with 7.4% having concomitant May–Thurner syndrome (MTS). In these patients, the classic form of MTS (compression of the left common iliac vein by the right common iliac artery against the lumbar spine) was either a pre-existent anomaly [52], secondary to the myoma [53,54,55,56], or was a combination of the two [8]. Most patients presented with lower extremity pain and swelling (47.5%), followed by chest symptoms and/or dizziness/syncope (21.2%)—Table 2.

### 2.4. Characteristics of Fibroids

The fibroid volume was reported in two observational studies. The average weight of the resected fibroids was 321 g [40], while the average uterine weight was 656 g (50–7000 g) [43]. The median fibroid volume in the case reports and series was 2050.15 mL (75.5 mL to 26,150 mL) (Table 2). A study reported a higher incidence of DVT (11.5%) when the uterine weight was 1000 g or more [43]. Compression of the vasculature or the surrounding structures (ureters) was reported in 80% of the cases. The most common site of compression was the inferior vena cava (IVC) (32%), followed by the left iliac vessels (19.4%), and in particular, the left common iliac vein (57%). The location of the DVT in patients often corresponded with the side of compression or, when the compression was more proximal, affected the IVC and common iliac veins. Fibroids can also compress the intestines, leading to intestinal obstruction [57]. Rarely, mesenteric vein thrombosis and intestinal gangrene were reported in association with uterine fibroids [58].

### 2.5. VTE Risk Factors

Various hematological factors and comorbid conditions correlated with an increased risk of VTE in women with uterine fibroids. A lower mean hemoglobin [40,41] and higher platelet count [40,42] were associated with an increased risk of VTE. Other factors like non-steroidal anti-inflammatory drug (NSAID) use [40], iron supplementation [40,41], cancer, coronary artery disease, and congestive heart failure [41] were also noted to be significantly associated with VTE. Approximately 60% of the patients lacked traditional risk factors for VTE. The mean hemoglobin of patients with fibroid-associated VTE was 8.8 ± 3.6 g/dL. A few case reports showed erythrocytosis, with one patient developing cerebral vein thrombosis [59], another PE [60], and the third extramedullary hematopoiesis that led to intravascular thrombi in the upper extremity [61]. A thrombophilia workup was reported in 59 patients, revealing only five positive cases (homozygous MTHFR gene and heterozygous Factor V Leiden mutations, Protein C and S deficiency, HIT and LA hypercoagulability mutation, Inherited Protein S deficiency, and heterozygous PT20210Z gene mutation) [44,62,63,64]. Other traditional VTE risk factors noted for 37.6% of the patients included medications such as hormone therapies, tranexamic acid, tamoxifen, prolonged immobilization, obesity, venous insufficiency, heart failure, diabetes mellitus, cancer, pregnancy and puerperium, family history, and polysubstance abuse.

### 2.6. Management Strategies

Only two-thirds (66.6%) of the patients had a prior diagnosis of fibroids before they presented with VTE. The remaining patients were either diagnosed with fibroids at the time of their VTE presentation or only after their VTE symptoms persisted despite undergoing treatment. One study showed that if the underlying cause was not addressed, many of these patients would go on to develop chronic VTE, such as CTEPH or post-thrombotic syndrome (PTS) [5]. The cornerstone of the treatment of these patients is therapeutic anticoagulation; however, in our review, the use of anticoagulation was reported in 73% of the cases. IVC filter or interruption was used in 35% of the cases to prevent PE. Hysterectomy was the primary fibroid treatment in 62% of the cases, and myomectomy was performed in 13% of the patients. In 5% of the cases, uterine artery embolization was demonstrated as a safe alternative to managing fibroids [65,66,67]. Several cases (8.4%) had complications associated with the treatment of fibroid-associated VTE, which included severe menstrual bleeding, necessitating surgical intervention [45,68,69,70,71,72]. Hormones, which were used in 15.6% of the cases to manage heavy menstrual bleeding caused by fibroids, could lead to VTE.

## 3. Discussion

Age may have a role in modifying the risk of VTE in women with fibroids. Our review delineated studies showing increased VTE risk in particular age groups like under 45 years or perimenopause [41,42]. This underscores the need for age-stratified, large-scale prospective research to validate this association and guide targeted screening efforts.

Although data on race were incomplete, 63% of the cases with documented race were Black women, reinforcing earlier assertions about disproportionate fibroid burden and VTE risk in this population. Black women are disproportionately affected by uterine fibroids, both in terms of how often they occur and how severe they are. Research shows that by the age of 50, nearly four out of five Black women will develop fibroids. These fibroids tend to be larger and cause more intense symptoms, such as significant menstrual bleeding, anemia, and pain [1,73]. These clinical challenges can indirectly raise the risk of VTE. For example, Black women are more frequently treated with surgical procedures like myomectomy or hysterectomy, both of which are linked to a higher risk of VTE during the perioperative period. Furthermore, the severity of symptoms and the recovery process after surgery can result in longer periods of immobility, which is a recognized risk factor for VTE.

Recent studies also suggest that Black individuals may have a naturally higher baseline risk for blood clotting compared to other populations. For instance, higher average levels of coagulation proteins such as Factor VIII and von Willebrand Factor (VWF) have been observed in Black populations [74,75,76]. Notably, increased VWF levels are important because they facilitate platelet clumping, which can increase the likelihood of clot formation [77]. Differences in how blood vessels function have also been reported between racial groups. Black women, for example, may be more prone to endothelial dysfunction, which is marked by lower nitric oxide availability and heightened vascular inflammation. This state can make the blood more prone to clotting and heighten the risk of VTE [78].

Our review revealed a clear predominance of left-sided DVT (Figure 1b) in patients with uterine fibroids. This pattern aligns with the well-established pathophysiology of May–Thurner syndrome, where the left common iliac vein is compressed by the overlying right common iliac artery, leading to venous stasis and an increased risk of thrombosis on the left side [79]. Notably, 7.4% of the cases in our review had documented concomitant MTS, supporting the hypothesis that fibroids may either exacerbate a pre-existing anatomical predisposition or contribute to secondary compression of the iliac vasculature. This association is recognized as clinically significant, as it underscores the importance of targeted venous imaging, such as iliac vein ultrasound or magnetic resonance venography in patients presenting with unilateral leg swelling, particularly on the left side. The early identification of underlying venous compression can guide timely intervention and reduce the risk of thrombotic complications.

Interestingly, 60% of the patients had no traditional VTE risk factors, suggesting that fibroids alone, through mass effect, hormonal influence, and hematologic changes, may serve as primary etiologic agents. The data also highlighted anemia (mean Hgb 8.8 g/dL) and thrombocytosis as recurrent lab findings. These may contribute to hypercoagulability through erythropoietin-driven marrow activity and reactive thrombocytosis. Rare but serious complications such as erythrocytosis, extramedullary hematopoiesis, and even paradoxical embolism in patients with a patent foramen ovale (PFO) emphasize the multisystem impact fibroids can have [59,60]. This supports calls for routine CBC evaluation and PFO screening in selected high-risk patients.

Cases of phlegmasia cerulea dolens, compartment syndrome, intestinal obstruction, and even mesenteric vein thrombosis with intestinal gangrene point to underrecognized, severe outcomes of fibroid-associated VTE. These highlight the importance of timely diagnosis and surgical or endovascular intervention, especially in women presenting with extensive thrombosis or systemic symptoms.

There are no uniform guidelines for treating VTE associated with uterine fibroids. The common treatment strategy includes anticoagulation, thrombus removal, and removing the source of compression. The recommended approaches include early anticoagulation, typically with novel oral anticoagulants (NOACs) or low-molecular-weight heparin (LMWH) for less extensive DVT, and interventions like thrombolysis and thrombectomy for more extensive DVT or PE cases. LMWH is preferred over unfractionated heparin due to its prompt effectiveness and possibly lower hemorrhage risk [80]. Optimal perioperative anticoagulation is poorly studied in randomized controlled trials. It is generally recommended to discontinue warfarin five days before surgery and consider LMWH bridging for high-risk patients, stopping it 12 h before surgery, while unfractionated heparin should be stopped about 6 h before surgery. Patients on NOACs are usually advised to hold anticoagulation for 24 to 48 h prior to surgery. The duration of holding anticoagulation may be longer in patients with kidney diseases and abnormal renal function. The duration of anticoagulation after fibroid removal is also uncertain. Women with large fibroids, especially those with clear signs of anatomical compression, typically may need only short-term anticoagulation therapy for three months as the risk of recurrence may be low [81]. The management of anticoagulation in patients with heavy menstrual bleeding due to fibroids also presents a clinical challenge. It involves careful pre-surgical planning and mechanical prophylaxis to mitigate venous stasis perioperatively.

Another aspect of the management of these patients is the prevention of PE by the placement of an IVC filter. Approximately 30–50% of patients with proximal DVT may develop symptomatic PE [70]. A common preventive strategy is placing an IVC filter, which can be considered before hysterectomy, to mitigate the risk of PE resulting from the relief of outflow obstruction post-surgery [8]. However, there are concerns about the potential complications associated with IVC filters and retrieving temporary filters; therefore, this strategy is considered controversial in contemporary practice [82]. In scenarios where placing an IVC filter is challenging due to uterine mass compression, alternative placements, such as a suprarenal position via a jugular approach, are considered [83].

The surgical removal of fibroids by hysterectomy or myomectomy is a primary management strategy in those VTE patients where fibroid-related venous compression is deemed to be the etiology of DVT. However, it is recommended to postpone surgery for a minimum of three months to ensure that embolization risks of the thrombus are minimal, or else consider IVC filter placement if surgery is necessary within four weeks [81]. An innovative approach involving a staged venous thrombectomy was used to treat iliofemoral DVT in the presence of a large uterine fibroid. The method combined an urgent IVC filter insertion before mechanical thrombectomy, followed by a hysterectomy to relieve external venous compression. A venogram plus angioplasty or stenting was then performed for residual stenosis [82]. It is important to note that failure to remove the source of extrinsic compression, such as large fibroids, can lead to recurrent thrombosis [5].

Recently, in some cases, uterine artery embolization has been used effectively as a less invasive alternative to hysterectomy to reduce uterine volume, facilitating DVT resolution [67]. This method is especially beneficial for heavy bleeding due to fibroids, allowing continued anticoagulation therapy. Ulipristal acetate (an anti-progesterone) may effectively stop fibroid-related bleeding without increasing the risk of thrombosis and notably reducing the uterine volume [84]. Other hormonal options include a levonorgestrel intrauterine system, progestins, and gonadotropin-releasing hormone analogs. Blood transfusions and iron supplementation may also be necessary to manage anemia in these patients, particularly when heavy bleeding is seen with anticoagulation use [81].

## 4. Future Direction

Significant knowledge gaps remain in understanding the complex relationship between uterine fibroids and VTE, particularly regarding underlying mechanisms, risk factors, and optimal management strategies. While mechanical compression of pelvic veins by large fibroids is a recognized contributor to VTE, broader physiological influences such as age-related changes, alterations in blood flow, anemia-induced hypercoagulability, and thrombocytosis require further investigation.

Race has also been shown to impact fibroid pathogenesis, with Black women experiencing earlier onset, larger fibroids, and more severe symptoms than white women [85]. However, the implications of these racial differences on the VTE risk are not well understood. The elevated VTE risk observed among Black women may reflect a combination of clinical and biological factors, including higher fibroid burden, more frequent surgical intervention, and differences in coagulation profiles, such as elevated von Willebrand factor levels.

Given the limited availability of fibroid diameter and uterine weight data in our study, conclusions regarding their role in VTE risk must be interpreted with caution. Future studies should stratify patients based on fibroid characteristics such as size, location, and uterine weight to allow for more accurate risk assessment and to support the development of targeted screening protocols.

The current understanding that large fibroids can obstruct venous flow and lead to VTE likely oversimplifies a multifactorial process. Individual variations in clinical presentation and outcome complicate the identification of high-risk patients, making it challenging to develop effective preventive strategies. For example, fibroids in postmenopausal women may undergo changes like calcification, which could alter their risk profiles [56,57].

Larger, prospective studies across diverse populations are essential to clarify the incidence, pathophysiology, and risk factors for VTE in patients with fibroids. Delays in diagnosis and management have resulted in severe complications such as pulmonary embolism, CTEPH, and paradoxical embolic strokes. Management strategies including anticoagulation, surgical or endovascular intervention, and timely fibroid treatment are currently based on case reports and expert opinion, leaving questions around optimal timing and approach, especially in patients with concurrent bleeding risks.

Standardized protocols are urgently needed to guide screening and management. These should emphasize the routine monitoring of fibroid growth and anatomical effects, particularly in patients presenting with symptoms such as leg swelling. Clinicians should maintain a low threshold for evaluating lower extremity deep vein thrombosis in women with large fibroids. Additionally, comprehensive risk assessments should account for other contributing factors to VTE to support proactive, individualized care.

Ultimately, future research must focus on refining risk stratification tools, addressing racial disparities, and developing evidence-based guidelines. Advancing our understanding of this intersection between fibroids and VTE will be critical to improving outcomes and safeguarding the health of women affected by uterine fibroids.

## 5. Conclusions

Uterine fibroids, though benign, can lead to serious vascular complications such as venous thromboembolism (VTE) through mechanisms involving mass effect, hematologic changes, and hormonal influences. Our review underscores a significant but underrecognized association between fibroids and VTE, especially in younger women and those of African descent. The predominance of left-sided DVT and frequent findings of vascular compression highlight the importance of targeted imaging and individualized risk assessment. Current evidence, primarily from case reports and limited observational studies, points to the need for heightened clinical awareness, early diagnosis, and multidisciplinary management strategies balancing thrombosis and bleeding risks. Prospective studies and clinical guidelines are urgently needed to optimize care for women affected by this complex overlap of conditions. The limitations of this review include the reliance on case reports, case series, and a limited number of observational studies, which are inherently subject to selection and publication bias. The absence of large-scale, prospective, and controlled studies limits the ability to establish causality and generalize findings to broader populations. Moreover, incomplete reporting of race, comorbidities, and long-term outcomes in many studies restricts a comprehensive understanding of the full clinical spectrum and prognostic implications. Future research should focus on well-designed cohort studies and registries to better define the incidence, pathophysiology, and optimal management strategies for fibroid-associated VTE. Until such data are available, heightened clinical awareness and individualized, multidisciplinary care remain essential.

## Figures and Tables

**Table 1:** Salient features of observational studies.

Author	Country	Patient Population	Sample Size	Time Period	Prevalence	Incidence	Comments
Latif et al., 2020 [40]	United States	Patients diagnosed with fibroids	6095	2015–2019	2.2% ^a^	n/a	
Huang et al., 2018 [41]	Taiwan	Female patients (>18y) with VTE	2282	2000–2013	n/a	n/a	Risk of VTE in fibroids: OR = 1.547 (95% CI: 1.27–1.88), *p* < 0.0001
Shiota et al., 2011 [42]	Japan	Patients diagnosed with fibroids undergoing hysterectomy	361	2003–2009	n/a	n/a	DVT rate for uterine weight <1000 g = 3% DVT rate for uterine weight > = 1000 g = 11.5%
Fletcher et al., 2009 [43]	Kingston, Jamaica	Adult women diagnosed with VTE	438	1999–2004	n/a	n/a	Risk of VTE in fibroids: OR = 3.75 (95% CI: 2.92–4.78), *p* = 0.0001

^a^: P revalence of VTE in fibroids.

**Table 2:** Salient features of case series and case reports.

Characteristics		Findings
Mean age (n = 93)		43.6 ± 7.8 years
Mean BMI (body mass index (n = 14)		26.7 ± 6.5 kg/m^2^
Mean hemoglobin (n = 42)		8.8 ± 3.6 g/dL
Race (n = 35)	Caucasian	7 (20%)
	African American/African/Afro-Caribbean/Caribbean	22 (62.9%)
	Asian	6 (17.1%)
Location of VTE (n = 97)	Proximal DVT (popliteal, femoral, iliac veins, IVC)	37 (38%)
	Distal DVT (peroneal, tibial, and muscular veins) with/without proximal DVT	6 (6.1%)
	DVT and PE	27 (27.6%)
	Acute PE only	6 (6.1%)
	CTED/CTEPH	7 (7.1%)
	Paradoxical embolus (PFO)	9 (9.2%)
	Others	5 (5.1%)
Side of DVT (n = 80)	Left	50 (63%)
	Right	19 (23.5%)
	Bilateral	11 (13.6%)
Symptoms on presentation ^✝^ (n = 99)	Lower extremity symptoms (swelling, pain)	47 (47.5%)
	Chest symptoms (pain, dyspnea), dizziness, syncope	21 (21.2%)
	Lower extremity and chest symptoms	11 (11.1%)
	Abdominal symptoms (pain, swelling)	9 (9.1%)
	Neurological symptoms	7 (7.1%)
	Others (headache, upper extremity symptoms, arterial thrombosis)	4 (4%)
Thrombophilia workup (n = 59)	Positive	5 (8.5%)
Known risk factors of VTE (n = 100)	Medications, immobilization, health conditions, addictions	38 (38%)
Signs of compression (n = 72)	Imaging/operative finding	57 (79.2%)
Management of VTE ^‡^ (n = 83)	IVC ^a^ filter/balloon occlusion	29 (35%)
	Anticoagulation	61 (73.5%)
	Thrombolysis	15 (18%)
	Thrombectomy/thromboembolectomy	15 (18%)
	Pulmonary thromboendarterectomy	7 (8.4%)
Management of fibroids (n = 83)	TAH ^b^	31 (37%)
	TAH ^b^ and BSO ^c^/BS ^d^/oophorectomy	21 (25%)
	Myomectomy	11 (13%)
	Uterine artery embolization	4 (5%)
	Drugs	5 (6%)
	Surgery planned/refused treatment/lost to follow-up	11 (13%)
Prior diagnosis of fibroid (n = 48)		32 (66.6%)
Mean gestational age of uterus (n = 18)		21.6 ± 4.5 weeks
Mean weight of uterus as operative finding (n = 16)		2231.9 ± 1343.7 g
Mean largest diameter of fibroid (n = 26)		13.5 ± 6.0 cm
Median volume of fibroid (n = 23)		2050.15 mL (75.5–26,250)

‘n’ refers to the number of patients in which the particular characteristics were documented. Abbreviations: ^a^: inferior vena cava, ^b^: total abdominal hysterectomy, ^c^: bilateral salpingo-oophorectomy, ^d^: bilateral salpingectomy. ^✝^ Including readmissions. ^‡^ Many patients had combinations of these treatments for VTE.
